# ApoJ/Clusterin concentrations are determinants of cerebrospinal fluid cholesterol efflux capacity and reduced levels are associated with Alzheimer’s disease

**DOI:** 10.1186/s13195-022-01119-z

**Published:** 2022-12-26

**Authors:** Yi-An Ko, Jeffrey T. Billheimer, Nicholas N. Lyssenko, Alexandra Kueider-Paisley, David A. Wolk, Steven E. Arnold, Yuk Yee Leung, Leslie M. Shaw, John Q. Trojanowski, Rima F. Kaddurah-Daouk, Mitchel A. Kling, Daniel J. Rader

**Affiliations:** 1 Division of Translational Medicine and Human Research, Perelman School of Medicine, University of Pennsylvania, 11-125 Smilow Center for Translational Research, 3400 Civic Center Blvd, Philadelphia, PA 19104-5158 USA; 2 Alzheimer’s Center at Temple, Department of Neural Sciences, Lewis Katz School of Medicine at Temple University, Philadelphia, PA 19140 USA; 3 Department of Psychiatry and Behavioral Sciences, Duke University, Durham, NC 27708 USA; 4 Department of Neurology, University of Pennsylvania, Philadelphia, PA 19104 USA; 5 Department of Neurology, Massachusetts General Hospital, Harvard Medical School, Boston, MA 02115 USA; 6 Penn Neurodegeneration Genomics Center, Department of Pathology and Laboratory Medicine, Perelman School of Medicine, University of Pennsylvania, Philadelphia, PA 19104 USA; 7 Department of Pathology and Laboratory Medicine, Perelman School of Medicine, University of Pennsylvania, Philadelphia, PA 19104 USA; 8 Duke Institute for Brain Sciences, Duke University, Durham, NC 27708 USA; 9 Department of Medicine, Duke University, Durham, NC 27708 USA; 10 Department of Geriatrics and Gerontology, New Jersey Institute for Successful Aging, Rowan University School of Osteopathic Medicine, 42 E. Laurel Rd., Suite 1800, Stratford, NJ 08084 USA; 11 Department of Psychiatry, Perelman School of Medicine, University of Pennsylvania, Philadelphia, Pennsylvania USA

## Abstract

**Background:**

Alzheimer’s disease (AD) shares risk factors with cardiovascular disease (CVD) and dysregulated cholesterol metabolism is a mechanism common to both diseases. Cholesterol efflux capacity (CEC) is an ex vivo metric of plasma high-density lipoprotein (HDL) function and inversely predicts incident CVD independently of other risk factors*.* Cholesterol pools in the central nervous system (CNS) are largely separate from those in blood, and CNS cholesterol excess may promote neurodegeneration. CEC of cerebrospinal fluid (CSF) may be a useful measure of CNS cholesterol trafficking. We hypothesized that subjects with AD and mild cognitive impairment (MCI) would have reduced CSF CEC compared with Cognitively Normal (CN) and that CSF apolipoproteins apoA-I, apoJ, and apoE might have associations with CSF CEC.

**Methods:**

We retrieved CSF and same-day ethylenediaminetetraacetic acid (EDTA) plasma from 108 subjects (40 AD; 18 MCI; and 50 CN) from the Center for Neurodegenerative Disease Research biobank at the Perelman School of Medicine, University of Pennsylvania. For CSF CEC assays, we used N9 mouse microglial cells and SH-SY5Y human neuroblastoma cells, and the corresponding plasma assay used J774 cells. Cells were labeled with [^3^H]-cholesterol for 24 h, had ABCA1 expression upregulated for 6 h, were exposed to 33 μl of CSF, and then were incubated for 2.5 h. CEC was quantified as percent [^3^H]-cholesterol counts in medium of total counts medium+cells, normalized to a pool sample. ApoA-I, ApoJ, ApoE, and cholesterol were also measured in CSF.

**Results:**

We found that CSF CEC was significantly lower in MCI compared with controls and was poorly correlated with plasma CEC. CSF levels of ApoJ/Clusterin were also significantly lower in MCI and were significantly associated with CSF CEC. While CSF ApoA-I was also associated with CSF CEC, CSF ApoE had no association with CSF CEC. CSF CEC is significantly and positively associated with CSF Aβ. Taken together, ApoJ/Clusterin may be an important determinant of CSF CEC, which in turn could mitigate risk of MCI and AD risk by promoting cellular efflux of cholesterol or other lipids. In contrast, CSF ApoE does not appear to play a role in determining CSF CEC.

**Supplementary Information:**

The online version contains supplementary material available at 10.1186/s13195-022-01119-z.

## Introduction

Alzheimer’s disease (AD), the most common cause of dementia, is characterized by the abnormal accumulation of amyloid-beta (Aβ) and hyperphosphorylated tau proteins, synaptic and neuronal dysfunction, neuroinflammation, and brain degeneration. Importantly, the brain is the most lipid-rich organ in the body and depends on tight regulation of lipid metabolism and transport to maintain proper neural signaling and cognitive function. This is particularly pertinent to AD, because increased cellular cholesterol content can increase Aβ production [1] and changes in cellular ceramides have demonstrable effects on Aβ production and aggregation into plaques [2–4]. Extensive genetics studies of AD have identified a number of genes that encode proteins associated with lipid metabolism, including apolipoproteins E and J and cellular lipid transporters ABCA1 and ABCA7 [5]. However, there is a distinct lack of information on lipid metabolism in the human brain and its relationship to AD.

Cholesterol biosynthesis occurs in all cells within the central nervous system (CNS) and some cell types can actively take up cholesterol from the extracellular space [6, 7]. Experimental evidence suggests that excessive cholesterol in neurons, astrocytes, and microglia promotes amyloid-beta (Aβ) accumulation and Aβ-driven inflammation in (AD) [8, 9]. While brain cells can convert cholesterol to 24S-hydroxycholesterol, they otherwise must efflux excess cholesterol (and 24S-hydroxycholesterol and potentially other lipid species) to acceptors in the extracellular space.

Cerebrospinal fluid (CSF) contains at least three apolipoproteins that are known to be lipid transport proteins and could serve as extracellular acceptors of cellular cholesterol and other lipids in the brain. ApoA-I, the major protein in plasma high density lipoproteins (HDL), is not synthesized in the brain, and CSF ApoA-I is derived from the plasma and is in concentrations in CSF approximately 1% of those in plasma [10] (and Supplemental Table 1). ApoE and ApoJ (also known as Clusterin) are both synthesized in the brain.

The cholesterol efflux capacity (CEC) assay was first developed by the Rader lab, using plasma [11] in order to measure the capacity of circulating plasma HDL to remove cholesterol from cholesterol-loaded macrophages. The assay is dependent on both the source cells and the acceptor media. With respect to cardiovascular disease, the source cells are cholesterol-rich macrophages that are a high source of transporters (e.g., ABCA1, ABCG1, and SRB1), which aid in the transport of the hydrophobic cholesterol molecule out of the membrane. Ex vivo analysis of serum CEC has been shown to be inversely and independently correlated with CVD [12–15]. Recently, we have shown that reverse cholesterol transport (RCT) can be measured in vivo [16]. In serum, ApoA1-containing lipoproteins are the main acceptors and are much higher in concentration than ApoE or ApoJ. Because cellular cholesterol is thought to play a role in AD, we adapted the CEC assay to use neuronal cells as source and CSF as acceptor [17]. Unlike serum, the concentration of ApoA1 is similar to that of ApoE and ApoJ in CSF such that all three may play a major role in efflux. Initial studies have shown that among the cells and conditions used, neuronal cells are a less important source cell than microglia, showing less efflux to ApoA1 and HDL [17].

We hypothesized that CSF CEC may be inversely associated with AD: high CSF CEC may facilitate cellular efflux of cholesterol (or other lipids) in a manner that is protective against AD, while low CSF CEC may have an opposite effect, potentially contributing to Aβ accumulation and the development of amyloid plaques in AD. We adapted our plasma CEC assay for CSF and showed that it is reproducible using several cell lines relevant to the CNS [17]. In that study, we found strong correlations between cholesterol efflux to CSF from the mouse microglial N9 cell line in our laboratory for assessing CEC in blood-based fluids. Here, we used this assay to measure CSF (and plasma) CEC in patients with AD, with mild cognitive impairment (MCI), and in cognitively normal (CN) controls, and also measured lipids, apolipoproteins, and known AD biomarkers in the same CSF samples.

## Methods

### Study design

CSF and plasma samples were obtained from the Biosample Repository of the Center for Neurodegenerative Disease Research (CNDR) at the Perelman School of Medicine (PSOM), University of Pennsylvania (Philadelphia, PA, USA). This case-control study examined participants enrolled in a prospective longitudinal study under the auspices of the National Institute on Aging (NIA)-funded Alzheimer’s Disease Clinical Center (ADCC) at PSOM. The participants were recruited at the Penn Memory Center, PSOM, and the Maria de los Santos Health Center (Philadelphia, PA, USA), following written informed consent under approval of the University of Pennsylvania Institutional Review Board.

Cases were classified as AD or MCI based on standard diagnostic criteria according to procedures established for ADCCs and the National Alzheimer’s Coordinating Center (NACC) [18–21]. From this cohort, we identified a subset of 127 CSF and plasma samples obtained on the same day from 112 unique participants and other CSF biomarker data (AD = 42, MCI = 20, and CN = 50). Four subjects were further removed due to major disruption of the blood brain barrier as adjudged by albumin and ApoB serum to CSF ratio (2 AD and 2 MCI samples, AD = 40, MCI = 18, and CN = 50). The total number of subjects used in this study is 108.

Subjects were selected from the Integrated Neurodegenerative Diseases Database (INDD) of the CNDR, using the web-based query tool INQUERY developed at CNDR. Neuropsychological testing was conducted including the Mini-Mental State Examination (MMSE) and/or tests of frontal executive function, memory, language, praxis, visuospatial construction, motor performance, mood, and function, based on the NACC Uniform Data Set (UDS) [21–23]. Details for CSF samples collection and for standardized Luminex assay for amyloid-beta (Aβ_1-42_), total tau (TTau), and phosphorylated tau (pTau) at the threonine 181 are available in previous publications [24–26].

Plasma and CSF aliquots (0.5 mL) were retrieved from the UPenn biorepository, coded to allow assays to be performed without knowledge of subject characteristics, and transported or shipped on dry ice to the respective laboratories for biochemical analysis.

The criteria for selecting CSF and plasma samples from the biobank were as follows: (1) subject evaluated at Penn Memory Center; (2) diagnosis of AD, MCI, or CN; and (3) availability of CSF and plasma from the same date.

### Biochemistry

Plasma cholesterol (Roche), ApoA-I (Roche), ApoB (Roche), and ApoE (Kamiya) concentrations were assayed on a Cobas C311 (Roche) and plasma and CSF human serum albumin by ELISA (E88-129; Bethyl Laboratories, Inc.). CSF cholesterol and triglyceride phospholipid were determined by fluorescence assays (A12216 (Invitrogen) and MAK122 (Sigma-Aldrich), respectively). CSF ApoA-I (DAPA10 (R&D Systems, Inc.)), ApoB (ab108807 (Abcam)), ApoE (ab108813 (Abcam)), and ApoJ (DCLU00 (R&D Systems, Inc.)) were determined by ELISA.

### Cholesterol efflux capacity (CEC) assays

The CSF CEC assay using N9 microglial cells and SH-SY5Y neuroblastoma cells has been described previously [17]. Briefly, SH-SY5Y cells were maintained in DMEM/4.5 g/L D-glucose/4mM L-glutamine/110 mg/L sodium pyruvate, and N9 were maintained in RPMI 1640/2mM L-glutamine (both media from Life Technologies), supplemented with 10% FBS. In the CSF CEC assay, cells were seeded in 96 well plates and labeled with 2 μCi/mL [1,2-^3^H(N)] cholesterol overnight. On day 2, SH-SY5Y were treated with 2 μM T0901317, and N9 and J774 cells were treated with 0.3 mM 8-CPT-cAMP to upregulate ABCA1. Cells were exposed to 33 μl of individual CSF and cholesterol efflux was allowed to proceed for 2.5 h. Cell medium was counted upon removal of floating cells and cell lipids were extracted with isopropanol read in a scintillation counter. Cell cholesterol efflux was quantified as the percent of [^3^H]-cholesterol counts in the medium relative to the total counts in medium and cells. A reference CSF sample was included on each plate to decrease inter-assay variability, and CEC activity was calculated as a unitless ratio of cholesterol efflux to sample CSF normalized to the reference CSF.

A similar assay with J774 mouse macrophages was used to determine plasma CEC using 1% ApoB depleted plasma in place of CSF and is described in Khera et al. [15] and Horiuchi et al. [27].

### Statistical analysis

For demographics, categorical variables are in percentages, and continuous variables as means with standard deviations or medians with range. Demographic statistics were computed using ANOVA and chi-square test for the continuous variable and categorical variables, respectively. For the post hoc multiple comparisons, we used the Tukey honestly significant difference (HSD) test. The correlation of efflux capacity between different cell lines was assessed with Spearman’s correlation coefficients. Correlations with *P* < 0.05 were considered significant. CEC values were log-transformed, and the N9 and SH-SY5Y CEC were not significant for the Shapiro-Wilk normality test (*P* = 0.58 for N9 and *P* = 0.66 for SH-SY5Y). For the association analysis, linear regression models were used to predict CEC, including lipoproteins and CSF biomarkers. Age, sex, race, and CSF (or plasma) storage time were incorporated as covariates in all analyses. We assessed the contribution of predictors in multinomial logistic regression, and calculated *β*, the logistic regression coefficient that shows the direction and size of the relationship between predictor and diagnosis. Odds ratio (OR) is the ratio of the odds that are calculated as the exponent of *β* with 95% confidence interval. The association between plasma CEC and biomarkers were analyzed using the same model. All analysis was carried out in the R application (v 3.6.0).

## Results

### Study demographics

The characteristics of the study cohort are shown in Table 1. Among the 108 participants, 40 (37.0%) were diagnosed as AD, 18 (16.7%) as MCI, and 50 (46.3%) were CN. Mean age at lumbar puncture was 69.9 years. Fifty-three percent were female, and 9% were African American. There were no significant differences among the three groups except for the MMSE as expected. The prevalence of the ApoE ε4 allele was highest in the AD group (65%) and lowest in the CN group (24%) as expected. AD biomarker CSF Aβ_1-42_ levels were significantly reduced in AD, whereas total Tau (TTau) and phosphorylated Tau (pTau) were significantly increased in AD. MCI subjects were intermediate between CN and AD in all cases (Supplemental Figure 1; Table 2).

**Table 1:** Demographic and clinical characteristics on study subjects

	CN (***n*** = 50)	MCI (***n*** = 18)	AD (***n*** = 40)	***P***-value
**Age**				0.422
Mean (SD)	68.8 (9.95)	70.5 (5.94)	71.3 (9.37)	
Median [min, max]	68.2 [48.9, 88.1]	67.9 [62.9, 83.1]	72.7 [51.4, 86.9]	
**Sex**				0.044 *
Female	30 (58.8%)	4 (25.0%)	24 (58.5%)	
Male	21 (41.2%)	12 (75.0%)	17 (41.5%)	
**Race**				0.23
Black	7 (13.7%)	0 (0%)	2 (4.9%)	
White	44 (86.3%)	16 (100%)	38 (92.7%)	
Asian	0 (0%)	0 (0%)	1 (2.4%)	
**MMSE Total**				9.33E−18^***^
Mean (SD)	29.1 (1.20)	27.1 (1.83)	20.6 (5.78)	
Median [min, max]	30.0 [25.0, 30.0]	27.0 [23.0, 30.0]	22.0 [5.00, 30.0]	
Missing	1 (2.0%)	1 (6.2%)	1 (2.4%)	
**Education**				0.424
Mean (SD)	16.3 (2.75)	15.6 (3.33)	15.6 (2.93)	
Median [min, max]	16.0 [12.0, 20.0]	16.0 [9.00, 20.0]	16.0 [11.0, 20.0]	

*P*-values were calculated using ANOVA; significant *P*-values: *P < 0.05; **P < 0.01; ***P < 0.001; *CN* cognitive normal, *MCI* mid cognitive impairment, *AD* Alzheimer’s disease, *MMSE* Mini-Mental State Examination

**Table 2:** One-way ANOVA test of CSF cholesterol efflux capacity, lipids, lipoproteins, and known AD biomarkers

	CN (***n*** = 50)	MCI (***n*** = 18)	AD (***n*** = 40)	***F***-statistics	***P***-value
**Aβ**_**42**_**(pg/mL)**^a,b^					
Mean (SD)	281 (77.8)	214 (90.5)	180 (60.7)	21.57	1.42e−08 ***
Median [min, max]	266 [140, 449]	192 [105, 354]	169 [76.0, 405]		
**Total Tau (pg/mL)**^b^					
Mean (SD)	52.2 (19.0)	70.0 (47.2)	105 (65.5)	15.59	1.18e−06 ***
Median [min, max]	48.0 [16.8, 105]	59.5 [17.0, 195]	85.4 [30.0, 299]		
**Phosphorylated Tau (pg/mL)**^b^					
Mean (SD)	22.1 (15.1)	29.9 (20.4)	42.9 (27.4)	9.702	1.36e−04 ***
Median [min, max]	19.0 [0.0400, 67.7]	24.5 [7.00, 77.0]	37.0 [0.0800, 114]		
**Phosphorylated Tau/Aβ42 ratio**^a,b^					
Mean (SD)	0.0970 (0.0852)	0.180 (0.161)	0.267 (0.170)	16	8.72e−07 ***
Median [min, max]	0.0700 [0.00700, 0.422]	0.100 [0.0400, 0.530]	0.210 [0.0600, 0.599]		
**CEC—N9 microglial cells**					
Mean (SD)	1.14 (0.319)	1.00 (0.246)	1.06 (0.272)	2.508	0.0863
Median [min, max]	1.09 [0.660, 2.11]	0.960 [0.600, 1.50]	1.04 [0.600, 1.93]		
**CEC—SHY5Y neuroblastoma cells**^a^					
Mean (SD)	1.45 (0.571)	1.14 (0.474)	1.39 (0.473)	3.212	0.0442 *
Median [min, max]	1.36 [0.570, 2.93]	1.04 [0.460, 2.25]	1.44 [0.740, 2.91]		
**Cholesterol (ng/mL)**					
Mean (SD)	166 (42.4)	176 (68.0)	163 (32.9)	0.442	0.644
Median [min, max]	160 [77.0, 270]	166 [89.0, 377]	164 [87.0, 230]		
Missing	4 (7.8%)	0 (0%)	3 (7.3%)		
**PL (nM)**					
Mean (SD)	10900 (2130)	9540 (1720)	10400 (1700)	1.651	0.197
Median [min, max]	11000 [6380, 16900]	10100 [7170, 13100]	10400 [6830, 15600]		
**ApoA1 (ng/mL)**					
Mean (SD)	2280 (1560)	1700 (588)	2190 (1030)	2.208	0.115
Median [min, max]	1840 [438, 6810]	1450 [1030, 3300]	2100 [601, 6330]		
**ApoE (ng/mL)**^b^					
Mean (SD)	11000 (1680)	10900 (2130)	9810 (1890)	4.784	0.0103 *
Median [min, max]	10900 [7390, 16100]	11000 [7310, 15000]	9340 [5910, 14000]		
**ApoJ (ng/mL)**^a^					
Mean (SD)	9690 (4030)	7350 (2420)	9080 (3210)	3.158	0.0466 *
Median [min, max]	8890 [2940, 19800]	7240 [3040, 10900]	8800 [2270, 17700]		
Missing	1 (2.0%)	0 (0%)	2 (4.9%)		

[1] All *P*-values are derived from one-way ANOVA

[2] All continuous measures are reported as medians with min and max ranges, missing value counts, and corresponding percentage

[3] CSF denotes cerebrospinal fluid, PL phosphorylated lipids, ApoA1 apolipoprotein A-I, ApoE apolipoprotein E, ApoJ Clusterin, Abeta42 42 amino acid form of beta amyloid

[4] Cholesterol efflux capacity is expressed as a percentage of efflux in the sample, normalized to a reference sample

[5] The Tukey HSD test was performed post hoc. We used a, b, and c to represent a significant difference in each comparison. a: MCI vs CN, b:AD vs CN, and c: AD vs MCI

Significant *P*-values: *P < 0.05; **P < 0.01; ***P < 0.001

### CSF CEC, but not plasma CEC, is lower in MCI compared with controls

**Fig. 1:**
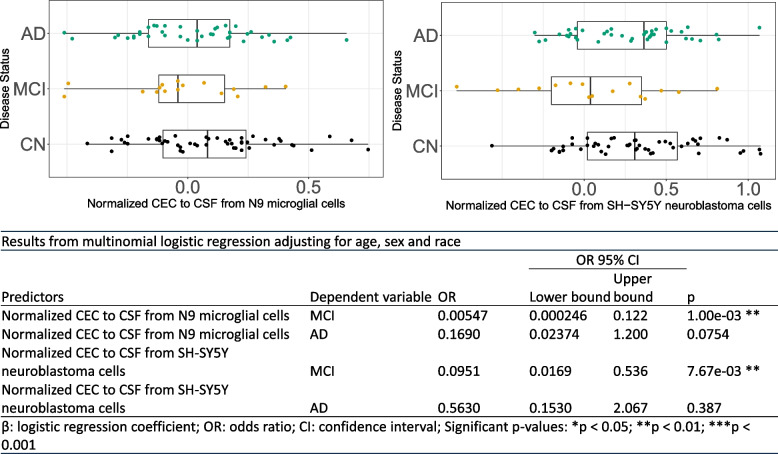
Multinomial logistic regression analyses for CSF cholesterol efflux capacity (CEC). The predictions were evaluated for MCI and AD groups against CN. CSF cholesterol efflux capacity was measured in CN (*n* = 50), MCI (*n* = 18), and AD (*n* = 40). The CEC values were normalized to a standard CSF sample that was run on each plate to account for inter-assay variability. We then log normalized the data prior to regression analysis. **A** Multinomial logistic regression analysis showed N9 CEC has a significant prediction of MCI. **B** Multinomial logistic regression analysis showed SHSY-5Y CEC has a significant prediction of MCI Detailed statistics are shown in the lower part of the figure. CN, cognitively normal; MCI, mild cognitive impairment; AD, Alzheimer’s disease; CSF, cerebrospinal fluid; CEC, cholesterol efflux capacity

CSF CEC was measured using both N9 microglial cells and SH-SY5Y neuroblastoma cells. The correlation of CSF CEC between the two cell types was moderate (correlation coefficient = 0.54, *P*-value = 1.3e−09) (Supplemental Figure 2A). Importantly, there was no association between CSF CEC and plasma CEC (Supplemental Figure 2B-C). CSF CEC was significantly lower in MCI subjects in SH-SY5Y neuroblastoma cells (*F* = 3.212, *P* = 0.0442). We further investigated the relationship between groups using multinomial logistic regression and observed a significant difference between MCI and CN for CECs from both cell types (Fig. 1 and Table 2). CSF cholesterol was significantly associated with CSF CEC from SH-SY5Y neuroblastoma cells (*B* = 26.0, *t* = 2.289, *P* = 0.0243) and from N9 microglial cells (*B* = 48.605, *t* = 2.883, *P* = 4.9e−03) (Supplemental Figure 3). CSF phospholipids were not significantly associated with CSF CEC in either cell type. There was no difference in CSF phospholipid, CSF cholesterol, plasma CEC, or cholesterol by diagnosis (Table 2 and Supplemental Table 1).

**Fig. 2:**
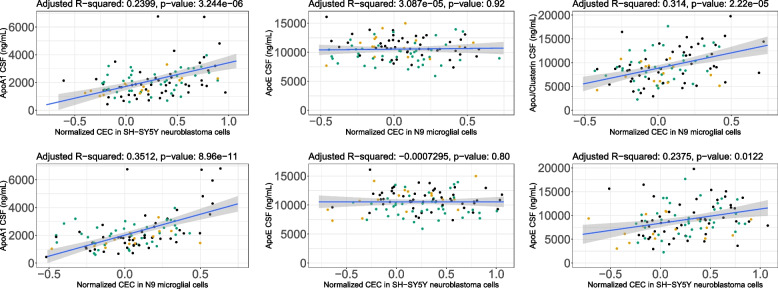
Multivariate linear regression of CSF CEC and apolipoproteins CSF. CSF cholesterol efflux capacity was measured in CN (*n* = 50), MCI (*n* = 18), and AD (*n* = 40). Association between ApoA1, ApoE, and ApoJ with both CEC in human microglial and neuronal cells (N9 and SH-SY5Y, respectively). Association between ApoA1 significantly associated with N9 microglial and SH-SY5Yneuronal cell CEC (**P < 8.96e−11 and ***P < 3.244e−06, respectively), ApoJ is significantly associated with N9 microglial and SH-SY5Yneuronal cell CEC (**P < 2.22e−05 and *P < 1.22e−02, respectively). ApoE is not associated with either CEC measurement

**Fig. 3:**
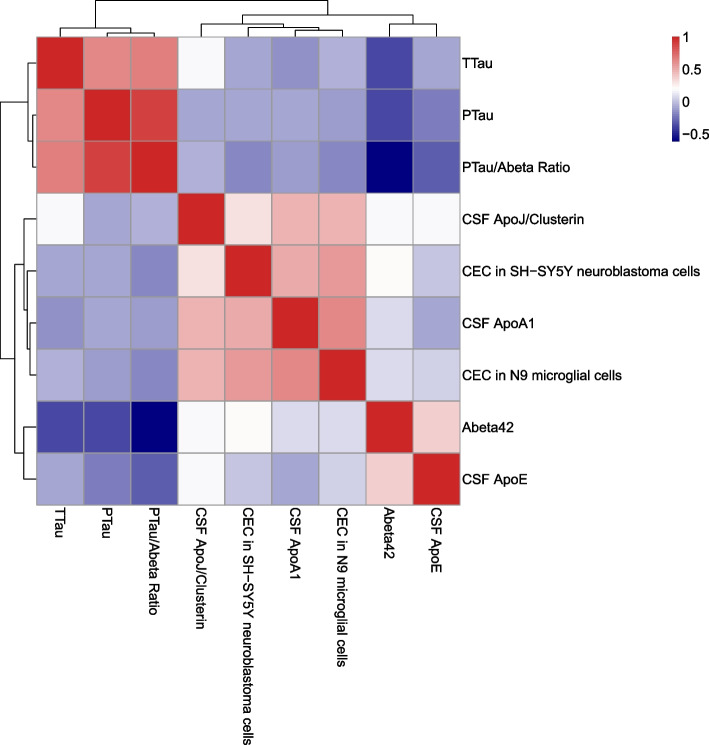
Heat map and hierarchical clustering of CSF CEC with apolipoproteins and biomarkers of AD. CSF CEC is closely associated with ApoA-I and Clusterin. Aβ_1-42_ is associated with ApoE, and pTau, TTau, and pTau/Aβ_1-42_ ratio

**Fig. 4:**
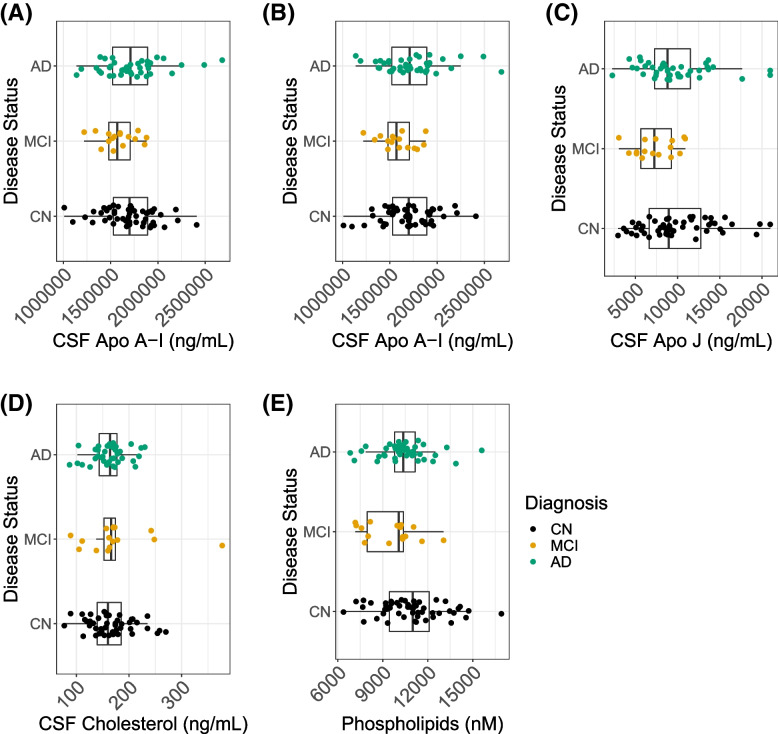
Multinomial logistic regression analyses for CSF apolipoproteins and AD diagnosis. Apolipoproteins were measured in CN (*n* = 50), MCI (*n* = 18), and AD (*n* = 40). **A** Apo A1 displayed significant prediction of MCI diagnosis. **B** ApoE is significantly lower in AD (**P < 0.01). **C** ApoJ (Clusterin) is significantly lower in MCI (**P < 0.01) AD denotes Alzheimer’s disease, CSF cerebrospinal fluid, ApoA1 apolipoprotein A-I, ApoE apolipoprotein E, ApoJ/Clusterin, PL phosphorylated lipid, and Aβ_1-42_ 42 amino acid form of beta amyloid

CSF ApoJ/Clusterin and ApoA-I were significantly associated with CEC and were lower in MCI compared to CN. ApoA-I, ApoE, and ApoJ were measured in CSF samples and ApoA-I and ApoE were measured in plasma samples (Table 2 and Supplemental Table 1). CSF ApoA-I and CSF ApoJ were each strongly correlated with both measures of CSF CEC, but ApoE was not (Fig. 2). Although we observed that CSF ApoJ and ApoA-I are closely correlated (Fig. 3), CSF ApoA-I and ApoJ were not significantly correlated with each other when we adjust for age, sex, and race (Supplemental Figure 4) CSF ApoA-I and ApoJ/Clusterin were both significantly lower in MCI than CN but not in AD, whereas CSF ApoE was significantly lower in AD subjects than in CN controls but not in MCI (Fig. 4 and Table 2). CSF ApoE levels were significantly lower in *APOE* ε4 allele carriers, and the AD group had significantly more ε4 allele carriers (Supplemental Figure 5). CSF ApoA-I had no correlation with plasma ApoA-I, and plasma ApoA-I was not different among CN, MCI, and AD subjects (Supplemental Table 1). Similarly, CSF and plasma ApoE levels were not correlated, and in contrast to CSF ApoE, plasma ApoE levels did not differ by diagnosis (Supplemental Table 1).

### CSF AD biomarkers

We measured Aβ_1-42,_ total Tau (TTau), and phosphorylated-Tau (pTau) in the same CSF samples. Aβ_1-42_ had a modestly significant association with CSF CEC in SH-SY5Y cells but not in N9 cells (Supplemental Figure 6). None of the other AD CSF biomarkers had an association with CSF CEC (Supplemental Figure 6). There was a strong and highly significant positive association between CSF Aβ_1-42_ and CSF ApoE and a strong negative association between pTau/Aβ_1-42_ ratio and CSF ApoE (Table 3; Supplemental Figure 6). Neither ApoA-I nor ApoJ had significant correlations with the AD biomarkers. We performed a pair-wise correlation analysis for all measurements made in the CSF (Fig. 3). ApoA-I, ApoJ, and the two CEC measurements clearly clustered together and were negatively associated with Tau and pTau. Aβ_1-42_ was positively correlated with ApoE and negatively with Tau and pTau.

**Table 3:** Results from multivariable linear regression adjusting for age, sex, and race

				95% CI for USC B				
Predictor	Dependent variable	USC B	Beta	Lower bound	Upper bound	*P*	*R*^2^	*R*^2^ adjusted
**CEC—N9**	**Aβ**_**1-42**_**(pg/mL)**	27.915	0.0935	− 35.5	91.330	0.385	0.03	− 0.029
**CEC—SH-SY5Y**	**Aβ**_**1-42**_**(pg/mL)**	39.792	0.237	5.71	73.870	0.0226 *	0.07216	0.01649
**ApoA-I (ng/mL)**	**Aβ**_**1-42**_**(pg/mL)**	1.97E−05	0.0629	− 4.67E−05	8.61E−05	0.5578	0.02574	− 0.03272
**ApoE (ng/mL)**	**Aβ**_**1-42**_**(pg/mL)**	0.0193	0.421	0.01	0.03	2.5e−05 ***	0.1822	0.1331
**ApoJ (ng/mL)**	**Aβ**_**1-42**_**(pg/mL)**	0.00439	0.202	− 4.04E−04	0.010	0.0722	0.0536	− 0.003175
**CEC—N9**	**Ttau (pg/mL)**	− 3.209	− 0.0181	− 37.171	30.753	0.852	0.206	0.158
**CEC—SH-SY5Y**	**Ttau (pg/mL)**	0.535	0.00536	− 18.134	19.204	0.955	0.210	0.163
**ApoA-I (ng/mL)**	**Ttau (pg/mL)**	3.48E−05	0.187	− 4.35E−09	6.96E−05	0.0500	0.240	0.195
**ApoE (ng/mL)**	**Ttau (pg/mL)**	− 1.28	− 0.0468	− 6.34	3.78	0.617	0.212	0.165
**ApoJ (ng/mL)**	**Ttau (pg/mL)**	0.00114	0.0881	− 1.45E−03	0.004	0.386	0.2161	0.1691
**CEC—N9**	**Ptau (pg/mL)**	− 6.827	− 0.0862	− 22.451	8.797	0.388	0.163	0.113
**CEC—SH-SY5Y**	**Ptau (pg/mL)**	− 1.047	− 0.0234	− 9.695	7.6	0.811	0.157	0.106
**ApoA-I (ng/mL)**	**Ptau (pg/mL)**	8.63E−06	0.104	− 7.72E−06	2.50E−05	0.297	0.1655	0.1154
**ApoE (ng/mL)**	**Ptau (pg/mL)**	− 0.00253	− 0.00	− 4.82E−03	− 2.37E−04	0.0309 *	0.1949	0.1466
**ApoJ (ng/mL)**	**Ptau (pg/mL)**	− 0.0008385	− 0.145	− 1.45E−03	3.73E−03	0.166	0.172	0.123
**CEC—N9**	**Ptau/Aβ**_**1-42**_**ratio**	− 0.0944	− 0.180	− 0.201	0.013	0.08298	0.1182	0.06417
**CEC—SH-SY5Y**	**Ptau/Aβ**_**1-42**_**ratio**	− 0.0413	− 0.139	− 0.101	0.018	0.170	0.107	0.0532
**ApoA-I (ng/mL)**	**Ptau/Aβ**_**1-42**_**ratio**	6.96E−08	0.126	− 4.35E−08	1.83E− 07	0.225	0.104	0.0493
**ApoE (ng/mL)**	**Ptau/Aβ**_**1-42**_**ratio**	− 2.39E−05	− 0.295	− 3.94E−05	− 8.38E−06	0.00288 **	0.169	0.118
**ApoJ (ng/mL)**	**Ptau/Aβ**_**1-42**_**ratio**	− 5.49E−06	− 0.142	− 1.38E−05	2.79E−06	0.191	0.106	0.0516

*USC* unstandardized regression coefficient, *SE* standard errors of the regression coefficients, *Sig* two-sided observed significance levels (*P*) for the *t* statistics. Significant *P*-values: *P < 0.05;**P < 0.01;***P < 0.001

## Discussion

Dysregulation of cholesterol metabolism in the brain has been associated with neurodegenerative disorders including AD. However, the exact mechanisms linking cholesterol metabolism and AD pathogenesis are not well understood and current understanding is sometimes conflicting. Prior work employing genome-wide association studies (GWAS) identified several genes involved in cholesterol movement as risk factors, including ApoE, ApoJ, ABCA1, and ABCA7. In this study, we used CSF from subjects with AD and MCI as well as cognitively normal controls to measure CSF cholesterol efflux capacity from two different cell types as well as lipids, apolipoproteins, and AD biomarkers. We studied whether these data were associated with AD/MCI diagnosis as well as with each other. We observed that both AD and MCI patients had significantly lower CSF Aβ_1-42_, increased CSF total and phosphorylated tau, lower CSF ApoE, and higher frequency of the *APOE* ε4 genotype, all of which have been reported and were positive controls for the experiment.

Our major findings are that reduced CSF CEC is a feature of MCI and early-stage AD, and the CSF CEC is strongly correlated with CSF ApoA-I and ApoJ, but not with CSF ApoE. CSF ApoA-I and ApoJ were significantly lower in MCI subjects than in controls, while ApoE is significantly lower in AD subjects compared to controls. Importantly, neither CSF CEC nor CSF ApoA-I were correlated with plasma CEC and ApoA-I; thus, plasma levels of ApoA-I and CEC cannot be used as surrogates for CSF.

Importantly, we measured CSF CEC with a novel approach using two different biologically relevant cell lines—the human microglial N9 cell line and the human neuronal SH-SY5Y cell line—by labeling the cells with radiolabeled cholesterol and then testing the capacity of the CSF samples to promote efflux. While previous studies have suggested decreased levels of CSF CEC in MCI and AD subjects [28, 29], they used different assays with murine J774 macrophages, CHO cells, and BHK cells that are less biologically relevant. Yassine et al. specifically focused on ABCG1-transporter-related efflux, using CHO or BHK cells transfected with ABCA1 transporter or J774 macrophages which have high ABCA1 relative to N9 and SH-SY5Y cells [28].

In our studies, the two independent CEC assays correlated well with each other. The most significant association with CSF CEC was with CSF ApoA-I and ApoJ, both of which are highly plausible acceptors of cellular cholesterol efflux. Indeed, ApoA-I and ApoJ are known to occur on the same lipoprotein particles in plasma [30]. One might postulate that an ApoA1/ApoJ complex is responsible for cholesterol efflux. However, in the CSF where ApoJ and ApoA1 are at similar concentrations, much of ApoJ is not associated with Apo A1 [10, 30, 31]. It is possible that ApoA-I and ApoJ may be important for promoting removal of cholesterol (and possibly other toxic lipids) from brain cells in a way that is protective against AD, although in CSF, ApoJ is also present on other lipoproteins as well as in soluble form [10].

The association between CSF ApoA-I and AD is not consistent in the literature [32–36]. Our results aligned with Saito et al. [36] that CSF ApoA-I is not associated with AD. Merched et al. reported a decrease in serum ApoA-I in AD subjects (with 59 CN and 98 AD) [37], and Smach et al. showed that serum ApoA-I is highly correlated with severity of AD [38], but we did not observe an association of plasma ApoA-I with AD or MCI. A notable finding of our study is that levels of CSF ApoJ (Clusterin) were significantly lower in MCI and trending in AD; they were also significantly positively associated with CSF CEC. Variants at the ApoJ/Clusterin locus have been significantly associated with AD by GWAS [39], suggesting that ApoJ/Clusterin has a causal role in influencing AD risk. Clusterin binds to Aβ_1-42_, and the complex is transported across the blood brain barrier (BBB) into the circulation through low-density lipoprotein receptor related protein 2 (LRP2) to promote clearance of Aβ peptides from the brain [40, 41]. More recently it has been shown that ApoJ/Clusterin can reverse neuroinflammation and memory loss in mice [42].

We previously reported that ApoJ/Clusterin predicts CSF CEC from microglia and astrocytes [17]. In our current analysis, we confirm that CSF ApoJ/Clusterin was significantly associated with CSF CEC but not with Aβ_1-42_, similar to a previous study [43]. Our results suggest that CSF ApoJ/Clusterin may protect against AD in part by promotion of lipid efflux from brain cells. Further studies are needed to investigate this hypothesis.

ApoE has a well-established role in AD [39], though the mechanisms remain unclear. CSF ApoE levels were lower in AD subjects, likely a reflection of the effect of the *APOE* ε4 allele in reducing CSF ApoE levels. Despite the fact that ApoE can act as an acceptor of cellular lipid efflux, we found no correlation of CSF CEC with CSF ApoE; we did, however, note a significant positive correlation between CSF ApoE and CSF Aβ_1-42._ In a previous study, the concentration of CSF ApoE was not associated with Aβ_1-42_ or clinical dementia diagnosis [44]. Another smaller study found CSF ApoE to be significantly associated with TTau, tau phosphorylated at Thr181 (pTau), and Aβ_1-42_ [45]. Our study suggests that ApoE does not influence AD risk through its effect on CSF CEC.

In summary, we found that CSF CEC measured using two different brain-relevant cell types was significantly lower in MCI patients than in cognitively normal controls. Both CSF ApoA-I and ApoJ (but not ApoE) were significantly associated with CEC. In addition, CSF ApoJ was significantly associated with MCI (lower) and trending in AD. Our major conclusions are that impaired CSF CEC, determined in part by CSF ApoJ concentrations, is a risk factor for MCI and AD. We speculate that factors regulating the synthesis and secretion of ApoJ in the brain influence the ability to promote efflux of cholesterol (and possibly other toxic lipids) from brain cells and therefore influence the risk of AD.

## Supplementary Information

**Additional file 1: Supplemental Figure 1.** Multinomial logistic regression analyses for CSF AD biomarkers and diagnosis. (A) Aβ_1-42_ is significantly lower in MCI and AD (**P = 9.43e-03 and ***P = 2.87e-06, respectively) (B) Total Tau is significantly higher in AD only (***P = 1.58e-04) (C) Phosphorylated Tau is significantly higher in MCI and AD (*3.37e-02 and ***P = 5.91e-04, respectively).

**Additional file 2: Supplemental Figure 2.** Multivariate linear regression between CSF CEC from N9, SH-SY5Y, and J774 efflux. (A) Spearman correlation of N9 human microglial cell efflux and SH-SY5Y human neuroblastoma cell efflux. The two CSF CEC are strongly correlated (R = 0.54, ***P = 1.34e-09). (B) Spearman correlation of N9 human microglial cell efflux and plasma efflux is not significant (*R* = -0.0621, *P* = 0.7374). (C) Spearman correlation of SH-SY5Y human neuroblastoma cell efflux and plasma efflux is also not significant (*R* = 0.0749, *P* = 0.2205).

**Additional file 3: Supplemental Figure 3.** Multivariate linear regression between CSF cholesterol and phospholipids with CSF CEC. (A) Phospholipids measured in CSF (μM) are not associated with SH-SY5Y CEC or N9. (B) Cholesterol measured in CSF (μg/mL) showed strong association with both N9 human microglial cell efflux and SH-SY5Y human neuroblastoma cell efflux (***P = 4.9e-3 and ***P = 9.32e-03, respectively).

**Additional file 4: Supplemental Figure 4.** Multivariate linear regression between CSF apolipoproteins. (A-C) CSF apolipoproteins are not significantly associated with each other. (D) ApoE is positively associated with Aβ_1-42_ (***P<0.001).

**Additional file 5: Supplemental Figure 5.** (A) Association between ApoE genotype and AopE concentration in CSF. The x-axis presents the ApoE genotype dosage. Each additional *APOE* ε4 allele are assigned as a positive integer, while each ε2 allele acts as a negative integer. Ε3 allele does not have any effect on the equation. This genotype model shows that the *APOE* genotype has a significant effect on ApoE concentration in CSF (***P = 2.45e-11). (B) The occurrence of ε4 allele carriers is significantly higher in the AD group (chi-square test *P*-value = 0.01058).

**Additional file 6: Supplemental Figure 6.** Multivariate linear regression of AD biomarkers with CEC in CSF. (A) SH-SY5Y human neuroblastoma cell CEC significantly associated with increased Aβ_1-42_ (*P = 0.0226) but not N9 microglial cell CEC. (B) TTau is not significantly associated with either SH-SY5Y human neuroblastoma cell or N9 microglial cell CEC. (C) PTau is not significantly associated with either SH-SY5Y human neuroblastoma cell or N9 microglial cell CEC. (D) PTau/Aβ_1-42_ ratio is not significantly associated with either SH-SY5Y human neuroblastoma cell or N9 microglial cell CEC.

**Additional file 7: Supplemental Table 1.** Concentrations of apolipoproteins in plasma.

## Data Availability

All data are available through the SAGE Synapse database as part of the M2OVE-AD program.

## References

[1] Wolozin B (2004). Cholesterol and the biology of Alzheimer’s disease. Neuron.

[2] Zinser EG, Hartmann T, Grimm MOW (2007). Amyloid beta-protein and lipid metabolism. Biochim Biophys Acta.

[3] Jazvinšćak Jembrek M, Hof PR, Šimić G (2015). Ceramides in Alzheimer’s disease: key mediators of neuronal apoptosis induced by oxidative stress and Aβ accumulation. Oxidative Med Cell Longev.

[4] Di Paolo G, Kim T-W (2011). Linking lipids to Alzheimer’s disease: cholesterol and beyond. Nat Rev Neurosci.

[5] Jansen IE, Savage JE, Watanabe K, Bryois J, Williams DM, Steinberg S (2019). Genome-wide meta-analysis identifies new loci and functional pathways influencing Alzheimer’s disease risk. Nat Genet.

[6] Orth M, Bellosta S (2012). Cholesterol: its regulation and role in central nervous system disorders. Cholesterol.

[7] Björkhem I, Lütjohann D, Diczfalusy U, Ståhle L, Ahlborg G, Wahren J (1998). Cholesterol homeostasis in human brain: turnover of 24S-hydroxycholesterol and evidence for a cerebral origin of most of this oxysterol in the circulation. J Lipid Res.

[8] Lee CYD, Tse W, Smith JD, Landreth GE (2012). Apolipoprotein E promotes β-amyloid trafficking and degradation by modulating microglial cholesterol levels. J Biol Chem.

[9] Abramov AY, Ionov M, Pavlov E, Duchen MR (2011). Membrane cholesterol content plays a key role in the neurotoxicity of β-amyloid: implications for Alzheimer’s disease. Aging Cell.

[10] Koch S, Donarski N, Goetze K, Kreckel M, Stuerenburg HJ, Buhmann C (2001). Characterization of four lipoprotein classes in human cerebrospinal fluid. J Lipid Res.

[11] Khera AV, Cuchel M, de la Llera-Moya M, Rodrigues A, Burke MF, Jafri K (2011). Cholesterol efflux capacity, high-density lipoprotein function, and atherosclerosis. N Engl J Med.

[12] Rosenson RS, Brewer HB, Davidson WS, Fayad ZA, Fuster V, Goldstein J (2012). Cholesterol efflux and atheroprotection: advancing the concept of reverse cholesterol transport. Circulation.

[13] Saleheen D, Scott R, Javad S, Zhao W, Rodrigues A, Picataggi A (2015). Association of HDL cholesterol efflux capacity with incident coronary heart disease events: a prospective case-control study. Lancet Diabetes Endocrinol.

[14] Rohatgi A, Khera A, Berry JD, Givens EG, Ayers CR, Wedin KE (2014). HDL cholesterol efflux capacity and incident cardiovascular events. N Engl J Med.

[15] Khera AV, Chaffin M, Aragam KG, Haas ME, Roselli C, Choi SH (2018). Genome-wide polygenic scores for common diseases identify individuals with risk equivalent to monogenic mutations. Nat Genet.

[16] Cuchel M, Raper AC, Conlon DM, Pryma DA, Freifelder RH, Poria R (2017). A novel approach to measuring macrophage-specific reverse cholesterol transport in vivo in humans. J Lipid Res.

[17] Cipollari E, Szapary HJ, Picataggi A, Billheimer JT, Lyssenko CA, Ying G-S (2020). Correlates and predictors of cerebrospinal fluid cholesterol efflux capacity from neural cells, a family of biomarkers for cholesterol epidemiology in Alzheimer’s disease. J Alzheimers Dis.

[18] McKhann G, Drachman D, Folstein M, Katzman R, Price D, Stadlan EM (1984). Clinical diagnosis of Alzheimer’s disease: report of the NINCDS-ADRDA Work Group under the auspices of Department of Health and Human Services Task Force on Alzheimer’s Disease. Neurology.

[19] McKhann GM, Knopman DS, Chertkow H, Hyman BT, Jack CR, Kawas CH (2011). The diagnosis of dementia due to Alzheimer’s disease: recommendations from the National Institute on Aging-Alzheimer’s Association workgroups on diagnostic guidelines for Alzheimer’s disease. Alzheimers Dement.

[20] Petersen RC, Smith GE, Waring SC, Ivnik RJ, Tangalos EG, Kokmen E (1999). Mild cognitive impairment: clinical characterization and outcome. Arch Neurol.

[21] Beekly DL, Ramos EM, Lee WW, Deitrich WD, Jacka ME, Wu J (2007). The National Alzheimer’s Coordinating Center (NACC) database: the Uniform Data Set. Alzheimer Dis Assoc Disord.

[22] Morris JC, Weintraub S, Chui HC, Cummings J, Decarli C, Ferris S (2006). The Uniform Data Set (UDS): clinical and cognitive variables and descriptive data from Alzheimer Disease Centers. Alzheimer Dis Assoc Disord.

[23] Weintraub S, Salmon D, Mercaldo N, Ferris S, Graff-Radford NR, Chui H (2009). The Alzheimer’s Disease Centers’ Uniform Data Set (UDS): the neuropsychologic test battery. Alzheimer Dis Assoc Disord.

[24] Kang J-H, Vanderstichele H, Trojanowski JQ, Shaw LM (2012). Simultaneous analysis of cerebrospinal fluid biomarkers using microsphere-based xMAP multiplex technology for early detection of Alzheimer’s disease. Methods.

[25] Shaw LM, Vanderstichele H, Knapik-Czajka M, Figurski M, Coart E, Blennow K (2011). Qualification of the analytical and clinical performance of CSF biomarker analyses in ADNI. Acta Neuropathol.

[26] Wang L-S, Leung YY, Chang S-K, Leight S, Knapik-Czajka M, Baek Y (2012). Comparison of xMAP and ELISA assays for detecting cerebrospinal fluid biomarkers of Alzheimer’s disease. J Alzheimers Dis.

[27] Horiuchi Y, Ohkawa R, Lai S-J, Shimano S, Hagihara M, Tohda S, et al. Usefulness of apolipoprotein B-depleted serum in cholesterol efflux capacity assays using immobilized liposome-bound gel beads. Biosci Rep. 2019:39. 10.1042/BSR20190213. 10.1042/BSR20190213 PMC6443949 30867253

[28] Yassine HN, Feng Q, Chiang J, Petrosspour LM, Fonteh AN, Chui HC, et al. ABCA1-mediated cholesterol efflux capacity to cerebrospinal fluid is reduced in patients with mild cognitive impairment and Alzheimer’s disease. J Am Heart Assoc. 2016:5. 10.1161/JAHA.115.002886. 10.1161/JAHA.115.002886 PMC4802440 26873692

[29] Marchi C, Adorni MP, Caffarra P, Ronda N, Spallazzi M, Barocco F (2019). ABCA1- and ABCG1-mediated cholesterol efflux capacity of cerebrospinal fluid is impaired in Alzheimer’s disease. J Lipid Res.

[30] de Silva HV, Stuart WD, Duvic CR, Wetterau JR, Ray MJ, Ferguson DG (1990). A 70-kDa apolipoprotein designated ApoJ is a marker for subclasses of human plasma high density lipoproteins. J Biol Chem.

[31] Suzuki T, Tozuka M, Kazuyoshi Y, Sugano M, Nakabayashi T, Okumura N (2002). Predominant apolipoprotein J exists as lipid-poor mixtures in cerebrospinal fluid. Ann Clin Lab Sci.

[32] Slot RER, Sikkes SAM, Berkhof J, Brodaty H, Buckley R, Cavedo E (2019). Subjective cognitive decline and rates of incident Alzheimer’s disease and non-Alzheimer’s disease dementia. Alzheimers Dement.

[33] Johansson P, Almqvist EG, Bjerke M, Wallin A, Johansson J-O, Andreasson U (2017). Reduced cerebrospinal fluid concentration of apolipoprotein A-I in patients with Alzheimer’s disease. J Alzheimers Dis.

[34] Castaño EM, Roher AE, Esh CL, Kokjohn TA, Beach T (2006). Comparative proteomics of cerebrospinal fluid in neuropathologically-confirmed Alzheimer’s disease and non-demented elderly subjects. Neurol Res.

[35] Sattlecker M, Kiddle SJ, Newhouse S, Proitsi P, Nelson S, Williams S (2014). Alzheimer’s disease biomarker discovery using SOMAscan multiplexed protein technology. Alzheimers Dement.

[36] Saito K, Seishima M, Heyes MP, Song H, Fujigaki S, Maeda S (1997). Marked increases in concentrations of apolipoprotein in the cerebrospinal fluid of poliovirus-infected macaques: relations between apolipoprotein concentrations and severity of brain injury. Biochem J.

[37] Merched A, Xia Y, Visvikis S, Serot JM, Siest G (2000). Decreased high-density lipoprotein cholesterol and serum apolipoprotein AI concentrations are highly correlated with the severity of Alzheimer’s disease. Neurobiol Aging.

[38] Smach MA, Edziri H, Charfeddine B, Ben Othman L, Lammouchi T, Ltaief A (2012). Polymorphism in apoA1 influences high-density lipoprotein cholesterol levels but is not a major risk factor of Alzheimer’s disease. Dement Geriatr Cogn Dis Extra.

[39] Deming Y, Xia J, Cai Y, Lord J, Holmans P, Bertelsen S (2016). A potential endophenotype for Alzheimer’s disease: cerebrospinal fluid clusterin. Neurobiol Aging.

[40] Bell RD, Sagare AP, Friedman AE, Bedi GS, Holtzman DM, Deane R (2007). Transport pathways for clearance of human Alzheimer’s amyloid beta-peptide and apolipoproteins E and J in the mouse central nervous system. J Cereb Blood Flow Metab.

[41] DeMattos RB, O’dell MA, Parsadanian M, Taylor JW, JAK H, Bales KR (2002). Clusterin promotes amyloid plaque formation and is critical for neuritic toxicity in a mouse model of Alzheimer’s disease. PNAS.

[42] De Miguel Z, Khoury N, Betley MJ, Lehallier B, Willoughby D, Olsson N (2021). Exercise plasma boosts memory and dampens brain inflammation via clusterin. Nature.

[43] Wang J, Zhang X, Zhu B, Fu P (2020). Association of clusterin levels in cerebrospinal fluid with synaptic degeneration across the Alzheimer’s disease continuum. Neuropsychiatr Dis Treat.

[44] Minta K, Brinkmalm G, Janelidze S, Sjödin S, Portelius E, Stomrud E (2020). Quantification of total apolipoprotein E and its isoforms in cerebrospinal fluid from patients with neurodegenerative diseases. Alzheimers Res Ther.

[45] Martínez-Morillo E, Hansson O, Atagi Y, Bu G, Minthon L, Diamandis EP (2014). Total apolipoprotein E levels and specific isoform composition in cerebrospinal fluid and plasma from Alzheimer’s disease patients and controls. Acta Neuropathol.

