# Supplementary figures and images for: ApoJ/Clusterin concentrations are determinants of cerebrospinal fluid cholesterol efflux capacity and reduced levels are associated with Alzheimer’s disease
Source: Alzheimers Res Ther. 2022 Dec 26;14:194. doi: 10.1186/s13195-022-01119-z (PMC9791777; doi:10.1186/s13195-022-01119-z)

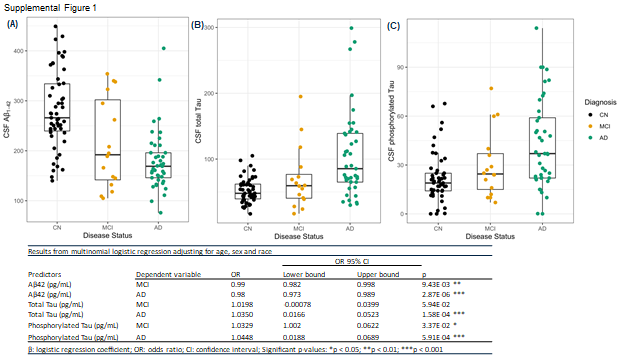

Supplement: Supplementary file 1 — Additional file 1: Supplemental Figure 1. Multinomial logistic regression analyses for CSF AD biomarkers and diagnosis. (A) Aβ1-42 is significantly lower in MCI and AD (**P = 9.43e-03 and ***P = 2.87e-06, respectively) (B) Total Tau is significantly higher in AD only (***P = 1.58e-04) (C) Phosphorylated Tau is significantly higher in MCI and AD (*3.37e-02 and ***P = 5.91e-04, respectively). [file 13195_2022_1119_MOESM1_ESM.docx]

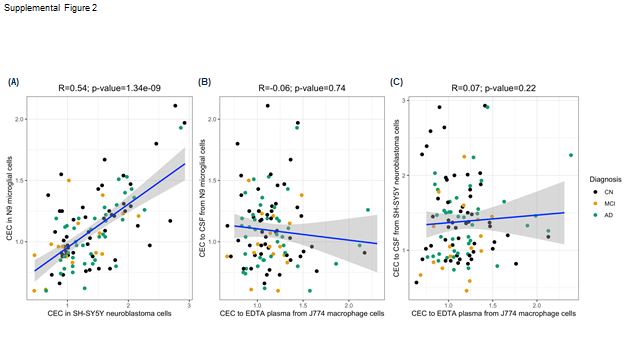

Supplement: Supplementary file 2 — Additional file 2: Supplemental Figure 2. Multivariate linear regression between CSF CEC from N9, SH-SY5Y, and J774 efflux. (A) Spearman correlation of N9 human microglial cell efflux and SH-SY5Y human neuroblastoma cell efflux. The two CSF CEC are strongly correlated (R = 0.54, ***P = 1.34e-09). (B) Spearman correlation of N9 human microglial cell efflux and plasma efflux is not significant (R = -0.0621, P = 0.7374). (C) Spearman correlation of SH-SY5Y human neuroblastoma cell efflux and plasma efflux is also not significant (R = 0.0749, P = 0.2205). [file 13195_2022_1119_MOESM2_ESM.docx]

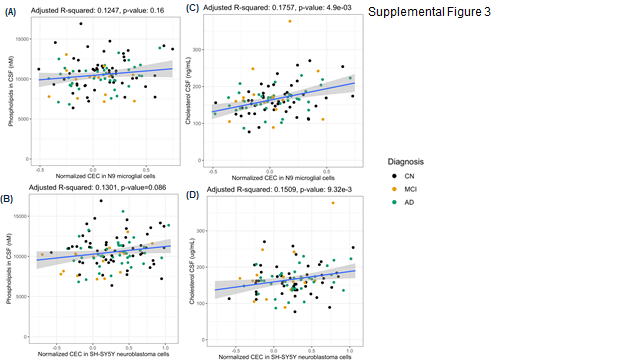

Supplement: Supplementary file 3 — Additional file 3: Supplemental Figure 3. Multivariate linear regression between CSF cholesterol and phospholipids with CSF CEC. (A) Phospholipids measured in CSF (μM) are not associated with SH-SY5Y CEC or N9. (B) Cholesterol measured in CSF (μg/mL) showed strong association with both N9 human microglial cell efflux and SH-SY5Y human neuroblastoma cell efflux (***P = 4.9e-3 and ***P = 9.32e-03, respectively). [file 13195_2022_1119_MOESM3_ESM.docx]

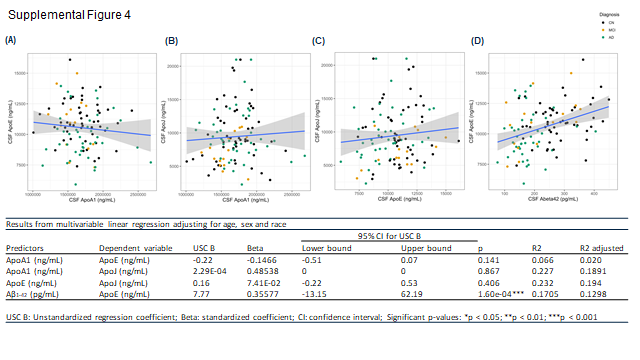

Supplement: Supplementary file 4 — Additional file 4: Supplemental Figure 4. Multivariate linear regression between CSF apolipoproteins. (A-C) CSF apolipoproteins are not significantly associated with each other. (D) ApoE is positively associated with Aβ1-42 (***P<0.001). [file 13195_2022_1119_MOESM4_ESM.docx]

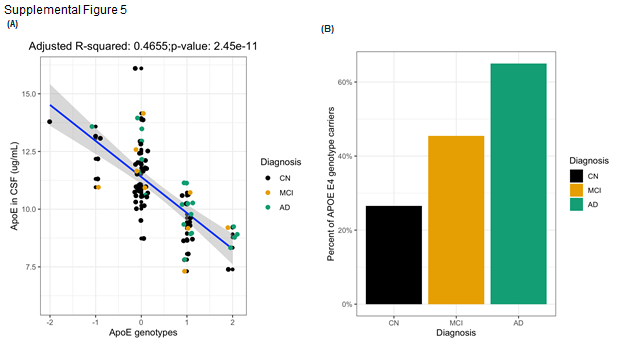

Supplement: Supplementary file 5 — Additional file 5: Supplemental Figure 5. (A) Association between ApoE genotype and AopE concentration in CSF. The x-axis presents the ApoE genotype dosage. Each additional APOE ε4 allele are assigned as a positive integer, while each ε2 allele acts as a negative integer. Ε3 allele does not have any effect on the equation. This genotype model shows that the APOE genotype has a significant effect on ApoE concentration in CSF (***P = 2.45e-11). (B) The occurrence of ε4 allele carriers is significantly higher in the AD group (chi-square test P-value = 0.01058). [file 13195_2022_1119_MOESM5_ESM.docx]

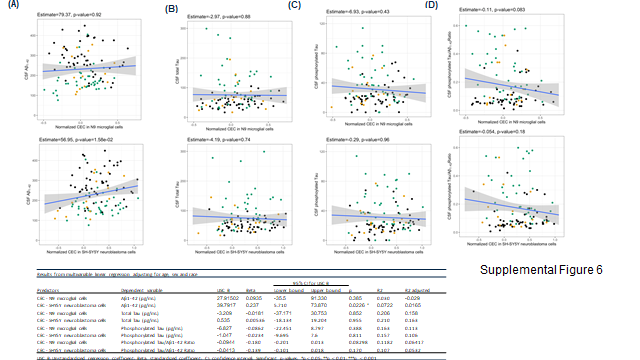

Supplement: Supplementary file 6 — Additional file 6: Supplemental Figure 6. Multivariate linear regression of AD biomarkers with CEC in CSF. (A) SH-SY5Y human neuroblastoma cell CEC significantly associated with increased Aβ1-42 (*P = 0.0226) but not N9 microglial cell CEC. (B) TTau is not significantly associated with either SH-SY5Y human neuroblastoma cell or N9 microglial cell CEC. (C) PTau is not significantly associated with either SH-SY5Y human neuroblastoma cell or N9 microglial cell CEC. (D) PTau/Aβ1-42 ratio is not significantly associated with either SH-SY5Y human neuroblastoma cell or N9 microglial cell CEC. [file 13195_2022_1119_MOESM6_ESM.docx]
