# Supplementary material for: ApoJ/Clusterin concentrations are determinants of cerebrospinal fluid cholesterol efflux capacity and reduced levels are associated with Alzheimer’s disease
Source: Alzheimers Res Ther. 2022 Dec 26;14:194. doi: 10.1186/s13195-022-01119-z (PMC9791777; doi:10.1186/s13195-022-01119-z)
Supplement: Supplementary file 7 — Additional file 7: Supplemental Table 1. Concentrations of apolipoproteins in plasma. [file 13195_2022_1119_MOESM7_ESM.docx]

| **Supplementary Table 1: Concentrations of apolipoproteins in plasma** | | |  | |  | |  | | |
| --- | --- | --- | --- | --- | --- | --- | --- | --- | --- |
|  | **CN (n=50)** | **MCI (n=18)** | | **AD (n=40)** | | **F-Statistics** | | | **p-value** |
| **CEC - J774 Cells** |  |  | |  | | 0.505 | | | 0.605 |
| Mean (SD) | 1.15 (0.269) | 1.11 (0.191) | | 1.19 (0.333) | |  |  |  |  |
| Median [Min, Max] | 1.14 [0.760, 2.24] | 1.11 [0.750, 1.42] | | 1.14 [0.860, 2.31] | |  |  |  |  |
| **Plasma ApoA1 (µg/mL)** |  |  | |  | | 2.208 | | | 0.115 |
| Mean (SD) | 170 (27.5) | 158 (18.4) | | 173 (31.3) | |  |  |  |  |
| Median [Min, Max] | 170 [101, 241] | 157 [122, 188] | | 171 [114, 268] | |  |  |  |  |
| **Plasma ApoE (µg/mL)** |  |  | |  | | 1.631 | | | 0.201 |
| Mean (SD) | 4.38 (1.62) | 4.12 (0.948) | | 3.86 (0.887) | |  |  |  |  |
| Median [Min, Max] | 4.10 [2.20, 11.8] | 4.20 [2.30, 5.40] | | 3.80 [2.30, 6.10] | |  |  |  |  |
| **Total Cholesterol in plasma (mg/dL)** |  |  | |  | | 0.655 | | | 0.521 |
| Mean (SD) | 199 (34.6) | 196 (46.5) | | 190 (41.0) | |  |  |  |  |
| Median [Min, Max] | 205 [122, 275] | 179 [152, 323] | | 178 [109, 343] | |  |  |  |  |
| **HDL-C in plasma (mg/dL)** |  |  | |  | | 1.916 | | | 0.152 |
| Mean (SD) | 61.6 (17.7) | 54.8 (10.6) | | 63.5 (18.4) | |  |  |  |  |
| Median [Min, Max] | 61.0 [27.0, 113] | 54.0 [34.0, 78.0] | | 62.0 [28.0, 109] | |  |  |  |  |
| [1] all p-values are derived from one-way ANOVA | | | | | |  | | |  |
| [2] Significant p-values: *p < 0.05; **p < 0.01; ***p < 0.001 | | | | |  | |  | | |
| [3] ApoA1 denotes apolipoprotein A-I, ApoE apolipoprotein E, HDL-C High-density lipoprotein cholesterol | | | | | | | |  |  |
